# Fabrication and Characterization of AlGaN-Based UV LEDs with a ITO/Ga_2_O_3_/Ag/Ga_2_O_3_ Transparent Conductive Electrode

**DOI:** 10.3390/nano9010066

**Published:** 2019-01-05

**Authors:** Hong Wang, Quanbin Zhou, Siwei Liang, Rulian Wen

**Affiliations:** 1 Engineering Research Center for Optoelectronics of Guangdong Province, School of Electronics and Information Engineering, South China University of Technology, Guangzhou 510640, China; zhouquanbin86@163.com (Q.Z.); 201620122572@mail.scut.edu.cn (S.L.); wrl_789@163.com (R.W.); 2 School of Physics and Optoelectronics, South China university of Technology, Guangzhou 510640, China; 3 Zhongshan Institute of Modern Industrial Technology, South China University of Technology, Zhongshan 528437, China

**Keywords:** transparent conductive electrode, Ga_2_O_3_, AlGaN-based ultraviolet light-emitting diode, transmittance, sheet resistance

## Abstract

We fabricated a complex transparent conductive electrode (TCE) based on Ga_2_O_3_ for AlGaN-based ultraviolet light-emitting diodes. The complex TCE consists of a 10 nm ITO, a 15 nm Ga_2_O_3_, a 7 nm Ag, and a 15 nm Ga_2_O_3_, forming a ITO/Ga_2_O_3_/Ag/Ga_2_O_3_ multilayer. The metal layer embedded into Ga_2_O_3_ and the thin ITO contact layer improves current spreading and electrode contact properties. It is found that the ITO/Ga_2_O_3_/Ag/Ga_2_O_3_ multilayer can reach a 92.8% transmittance at 365 nm and a specific contact resistance of 10^−3^ Ω·cm^2^ with suitable annealing conditions.

## 1. Introduction

AlGaN-based ultraviolet (UV) light-emitting diodes (LEDs) can achieve the full wavelength coverage of UVA (400–320 nm), UVB (320–280 nm) and UVC (280–200 nm) by changing Al content. As a result, AlGaN-based UV LEDs have attracted considerable attention and are seen as a promising lighting source for different applications in environmental cleaning, medicine, printing, microscopy and lighting [1,2,3,4,5,6]. However, the external quantum efficiency (EQE) of AlGaN-based UV LEDs is still much lower than that of the commercially available blue LEDs with an EQE close to 20% for UVA and <1% for UVC devices [7,8,9]. This phenomenon obstructs commercial applications of the AlGaN-based UV LEDs. Indium tin oxide (ITO) is widely used as transparent contact layers in traditional GaN-based blue and green LEDs. However, there is serious light absorption in the ITO in the ultraviolet band due to the band gap of ITO ranging from 3.5 eV to 4.3 eV [10,11]. Previous studies reported that doping metals in ITO would reduce the light absorption in near UV LEDs. The transmittance of ITO at wavelengths above 380 nm can reach about 90% by optimizing the thickness of metal and the annealing temperature [12,13,14,15]. But the transmittance of ITO still decreases rapidly when the wavelength becomes shorter. Thus, it is very urgent for a layer with higher transmittance in ultraviolet band to be able to replace the traditional ITO transparent conductive electrode (TCE) in UV LEDs.

Ga_2_O_3_, which has a bandgap from 4.9 eV to 5 eV, is an attractive alternative for TCE in UV LEDs because of its high transmittance in UV band [16,17,18]. In addition, a large size and high quality Ga_2_O_3_ thin film can be fabricated by single crystals synthesized by the melt growth method [19]. This material has been studied in the fields of metal semiconductor field effect transistors, metal oxide semiconductor field effect transistors and Schottky barrier diodes. However, the conductivity of Ga_2_O_3_ is very poor. Many approaches have been developed to promote the conductivity of Ga_2_O_3_. Orita Mi Hiramatsu H et al. improved the conductivity of β-Ga_2_O_3_ by doping In or Sn into Ga_2_O_3_ [16]. The (201)-oriented Sn-doped β-Ga_2_O_3_ films obtained a maximum conductivity of 8.2 S/cm (about 1.22 × 10^4^ Ω/sq). But it is still too low to be used as TCE in UV LED. Liu JJ et al. grew ITO thin films in Ga_2_O_3_ films and improved the sheet resistance and transmittance of Ga_2_O_3_/ITO films by adjusting the growth temperature and the thickness of ITO [17]. A sheet resistance of 323 Ω/sq and a transmittance at 280 nm of 77.6% can be achieved. Jae-kwan Kim et al. realized that the transmittance at 380 nm is 80.944% and the sheet resistance is 58.6 Ω/sq [20]. The Kie Young Woo group in Korea prepared the Ag/Ga_2_O_3_ model by learning the ITO/Ag/ITO model [21]. The contact characteristics and conductivity of the Ga_2_O_3_ films were improved by the Ag intercalation layer, and the transmittance at 380 nm and specific contact resistivity of the Ag/Ga_2_O_3_ thin film were 91% and 3.06 × 10^−2^ Ω·cm^2^ respectively.

In this paper, a complex TCE based on Ga_2_O_3_ is proposed to enhance the efficiency of UV LEDs. We prepared the complex Ga_2_O_3_-based TCE by depositing an ITO contact layer, a Ga_2_O_3_ layer, an Ag metal intercalation layer and another Ga_2_O_3_ layer in sequence, forming an ITO/Ga_2_O_3_/Ag/Ga_2_O_3_ multilayer. The resistance and transmittance ITO/Ga_2_O_3_/Ag/Ga_2_O_3_ multilayer with a different annealing temperature were studied and analyzed systematically. The sheet resistance of the ITO/Ga_2_O_3_/Ag/Ga_2_O_3_ multilayer was detected by four-point probe methods. The optical transmittance was measured by a UV/visible spectrophotometer. The surface roughness of these ITO/Ga_2_O_3_/Ag/Ga_2_O_3_ multilayer were measured by atomic force microscope (AFM). The X-ray photoelectron spectroscopy (XPS) and Auger electron spectroscopy (AES) measurements were also used to analyze the ITO/Ga_2_O_3_/Ag/Ga_2_O_3_ multilayer. Furthermore, we employed the ITO/Ga_2_O_3_/Ag/Ga_2_O_3_ multilayer as TCEs on 365 nm UV epitaxy in comparison to those with conventional ITO.

## 2. Materials and Methods

To investigate the influence of a Ag intercalation layer on the Ga_2_O_3_ layer, a Ga_2_O_3_/Ag/Ga_2_O_3_ (15 nm/7 nm/15 nm) multilayer was deposited on quartz substrates and then annealed at different conditions. The quartz substrates were first washed in acetone, isopropanol and deionized water and dried by nitrogen. After that, Ga_2_O_3_, Ag and Ga_2_O_3_ were sequentially deposited on the quartz substrates in magnetron sputtering equipment. In order to reduce the resistivity of the Ga_2_O_3_ layer but not affect its transmittance, the thickness of the Ag embedding interlayer and Ga_2_O_3_ were set to be 7 nm and 15 nm respectively. The Ga_2_O_3_ thin films were all deposited by RF magnetron sputtering of Ga_2_O_3_ (purity 99.99%) ceramic target, and the Ag thin film was deposited by direct current magnetron sputtering of the Ag target. The sputtering cavity was pumped to 5 × 10^−6^ Pa before the sputtering begin. The sputtering atmosphere was pure argon with the pressure of 5 mtorr. The rotation speed of the cavity substrate is 20 rpm. The temperature was controlled at about 35 °C ± 1 °C by feedback control heater during deposition. Afterwards, all the Ga_2_O_3_/Ag/Ga_2_O_3_ multilayer samples were annealed by a rapid thermal annealing (RTA) system at a different temperature and ambient. We used X-ray photoelectron spectroscopy (XPS) and Auger electron spectroscopy (AES) to analyze the element diffusion effect of the Ga_2_O_3_/Ag/Ga_2_O_3_ multilayer.

To further improve the contact between Ga_2_O_3_ and AlGaN-based UV epitaxy, we insert an ITO thin film below the Ga_2_O_3_/Ag/Ga_2_O_3_ multilayer as a contact layer. We prepared ITO/Ga_2_O_3_/Ag/Ga_2_O_3_ multilayer on quartz substrates. Before the deposition of Ga_2_O_3_/Ag/Ga_2_O_3_ multilayer, a 10 nm ITO was deposited on quartz substrates by RF magnetron sputtering of ITO (In_2_O_3_: 90 wt%, SnO_2_: 10 wt%) and then annealed by RTA. Subsequently, Ga_2_O_3_/Ag/Ga_2_O_3_ multilayer was deposited on the annealed ITO thin films and the whole ITO/Ga_2_O_3_/Ag/Ga_2_O_3_ multilayer was annealed again.

**Figure 1:**
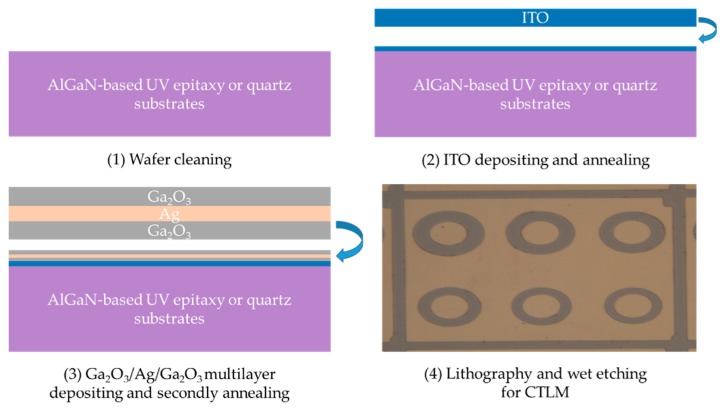
The procedures of ITO/Ga_2_O_3_/Ag/Ga_2_O_3_ multilayer and optical micrograph of contact surface on CTLM patterns.

Finally, we prepared ITO/Ga_2_O_3_/Ag/Ga_2_O_3_ multilayer on AlGaN-based UV epitaxy in the same method to study the specific contact resistance through the CTLM model. A 47 nm ITO thin film on quartz substrates and epitaxy was also prepared as reference, which was annealed at 600 °C for 1 min in a mixture of N_2_/O_2_ (200 sccm:35 sccm) ambient. The procedures of ITO/Ga_2_O_3_/Ag/Ga_2_O_3_ multilayer and optical micrograph of contact surface on CTLM patterns are shown in Figure 1.

## 3. Results

In order to study the influence of annealing conditions on the sheet resistance of Ga_2_O_3_/Ag/Ga_2_O_3_ multilayer, a series of Ga_2_O_3_/Ag/Ga_2_O_3_ multilayer on quartz substrates were annealed at different temperature and ambient. The annealing temperature changed from 400 °C to 600 °C with the annealing ambient changing from N_2_/O_2_ mixture and pure O_2_ ambient. As shown in Table 1, the sheet resistance increases with the decrease of annealing temperature. It is found that the Ga_2_O_3_/Ag/Ga_2_O_3_ multilayer could reach the lowest sheet resistance of 16.45 Ω/sq after being annealed at 600 °C for 1 min in an N_2_/O_2_ mixture ambient. The result means that the effect of Ag as the insertion layer is not obvious at low temperature, and the metal diffusion reaction is not sufficient. The metal insertion layer in the film can fully diffuse to the Ga_2_O_3_ layer and decrease the resistance value of the Ga_2_O_3_/Ag/Ga_2_O_3_ multilayer when the temperature reaches 600 °C. The resistance of the multilayer annealed at 600 °C in pure oxygen ambient is higher than that of the multilayer in an N_2_/O_2_ mixture annealing ambient. Besides, the higher the oxygen ratio in the annealing atmosphere, the higher the multilayer resistance value becomes. The reason for this is that metal oxides form and then affect the resistance of film [22,23].

**Figure 2:**
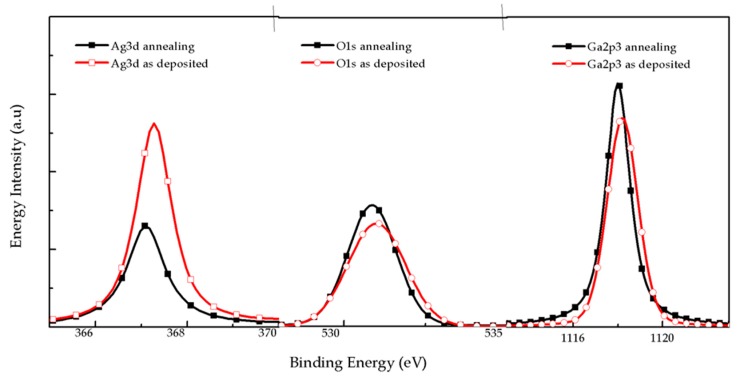
XPS spectrum for Ag3d, O1s and Ga2p_3_ of Ga_2_O_3_/Ag/Ga_2_O_3_ multilayer on quartz substrates.

Figure 2 and Table 2 show the XPS energy spectral of Ga_2_O_3_/Ag/Ga_2_O_3_ multilayer on quartz substrate before and after annealing at 600 °C for 1 min in N_2_/O_2_ mixture ambient. The energy intensity, peak value quantum-number vertex, high half-width and atomic fraction content of Ag3d, O1s, Ga2p_3_ were measured at the depth of about 10 nm of the multilayer. The open symbol and solid symbol in Figure 2 represent the energy intensity of the elements before and after annealing respectively. We can see that the energy value of element Ag3d is high, the quantum number per second is 13581 states/s, and the atomic fraction is 0.47 before annealing. After annealing, the energy intensity and the atomic ratio of Ag atom decrease relatively, which is 6651.8 counts/s and 0.29%, respectively. The energy and atomic ratio of Ga and O increase a little after annealing. The decrease of atomic ratio of Ag atom means that the process of annealing results in diffusion of Ag in the multilayer. Therefore, the sheet resistance of annealed Ga_2_O_3_/Ag/Ga_2_O_3_ multilayer decreases compared to that of the as-deposited sample due to the diffusion of internal elements.

**Figure 3:**
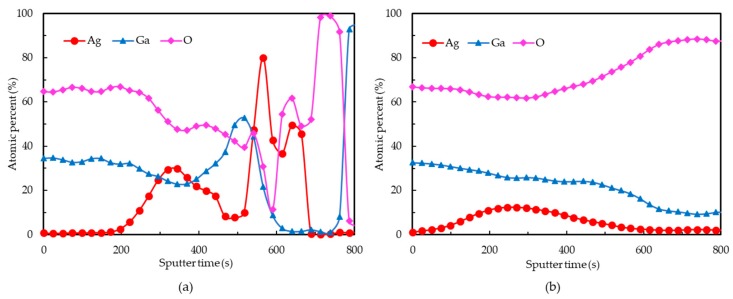
AES depth profiles of the Ga_2_O_3_/Ag/Ga_2_O_3_ multilayer on quartz substrates (**a**) before annealing and (**b**) after annealing.

In addition, to further identify the distribution of composition in Ga_2_O_3_/Ag/Ga_2_O_3_ multilayer, we analyzed the Ga_2_O_3_/Ag/Ga_2_O_3_ multilayer on quartz substrate using AES measurement. Figure 3 shows the AES depth profiles of the Ga_2_O_3_/Ag/Ga_2_O_3_ multilayer before and after annealing at 600 °C for 1 min in N_2_/O_2_ mixture ambient. For the multilayer before annealing, the atomic percent of Ag is low in the surface and increases after a specific sputter time, which means that the Ag do not diffuse into the multilayer. Since the Ga_2_O_3_ and quartz substrates have poor conductivity, the atomic percent will become random and fluctuant due to the charge accumulation effect when the sputter time increases. By contrast, the atomic percent of Ag increases at the beginning of sputtering and the Ag atoms distribute more evenly in the whole multilayer after annealing as shown in Figure 3b. This result demonstrates that the Ag will diffuses into the Ga_2_O_3_ layer during the annealing process, leading to the reduction of sheet resistance of the Ga_2_O_3_/Ag/Ga_2_O_3_ multilayer.

**Figure 4:**
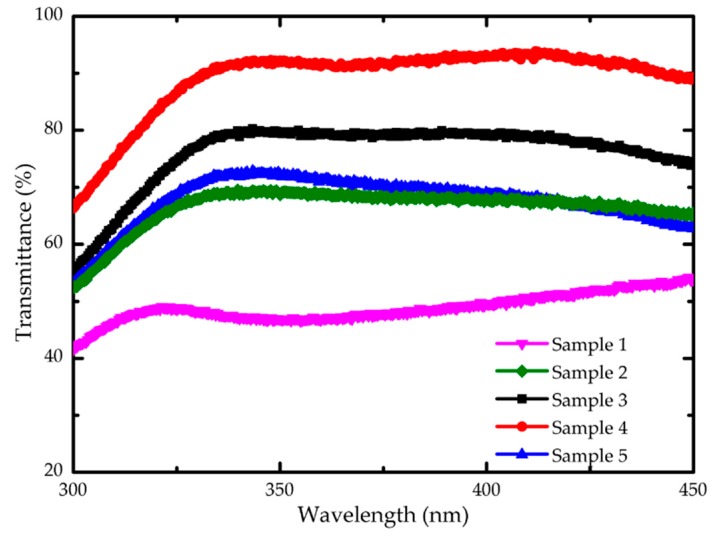
The transmittance curves of ITO/Ga_2_O_3_/Ag/Ga_2_O_3_ multilayer on quartz substrates after annealing.

Because of the bad contact property between Ga_2_O_3_ and p-GaN on epitaxial wafer, we insert a 10 nm ITO thin film below Ga_2_O_3_ as the contact layer. In order to optimal the transmittance and sheet resistance of the ITO/Ga_2_O_3_/Ag/Ga_2_O_3_ multilayer, we prepared five ITO/Ga_2_O_3_/Ag/Ga_2_O_3_ multilayer samples on quartz substrates and changed the annealing temperature as shown in Table 3. Among the five samples, sample 1 was not annealed. Sample 2 was annealed at 600 °C as a whole. For sample 3 to sample 5, the 10 nm ITO layer was firstly annealed at 550/600/650 °C respectively and then the whole ITO/Ga_2_O_3_/Ag/Ga_2_O_3_ multilayer were annealed at 600 °C. The annealing process of ITO and ITO/Ga_2_O_3_/Ag/Ga_2_O_3_ multilayer both maintained in N_2_/O_2_ (200 sccm:35 sccm) mixture ambient for 1 min. Figure 4 is the transmittance curves of five samples at range of 300 nm to 450 nm. It is obvious that sample 4, which was annealed at 600 °C at first and then at 600 °C again, has the highest transmittance of 92.68% at 365 nm and the lowest sheet resistance of 20.1 Ω/sq.

**Figure 5:**
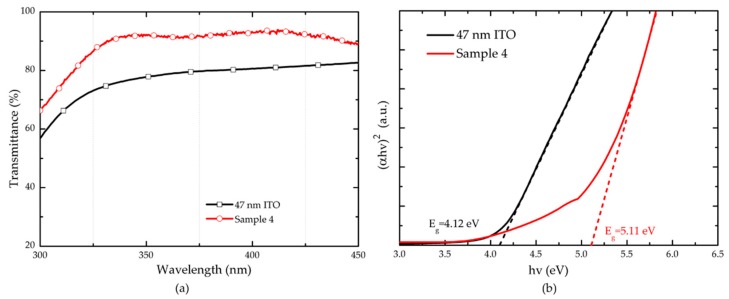
(**a**) Transmittance and (**b**) Energy bandgap of sample 4 and 47 nm ITO on quartz substrates.

In addition, we compared the transmittance and sheet resistance of sample 4 and a 47 nm ITO thin film on quartz substrate. The 47 nm ITO sample was annealed at 600 °C in N_2_/O_2_ (200 sccm:35 sccm) mixture ambient for 1 min. Figure 5a plots the transmittance curves of sample 4 and 47 nm ITO. The ITO/Ga_2_O_3_/Ag/Ga_2_O_3_ multilayer demonstrates better transmittance property than 47 nm ITO especially in UV range. To further understand the origin of this result, the optical bandgap Energy *Eg* of sample 4 and 47 nm ITO was calculated. The *Eg* can be extracted from the relation between (*αhv*)^2^ and *hv* according to the Equations (1) and (2), as follow:

(1) $$\alpha hv=C{\left(hv-E_{g}\right)}^{1/2}$$

(2) $$hv=\frac{hc}{\lambda_{i}}$$

The *Eg* can be obtained by extrapolating the linear (*αhv*)^2^ versus *hv* plots to the horizontal axis [24,25]. In Equations (1) and (2), *C* is a constant of direct transition, *α* is the light absorption coefficient, *hv* is the photon energy, *h* is Planck constant bright, *c* is the light speed, and *λ_i_* is the wavelength [24,26,27].

If the transmittance *T* at each *λ_i_* is known, the value of *α* at each *λ_i_* can be obtained by Equations (3) and (4), as follow:

(3) T=exp(−αd)

(4) $$\alpha =\frac{Ln\left(\frac{1}{T}\right)}{d}$$

where *d* is the thickness of films. Since we have measured the transmittance *T* of sample 4 and 47 nm ITO, the curves of (*αhv*)^2^ as a function of *hv* can be obtained as shown in Figure 5b. The optical Energy bandgap *Eg* of sample 4 is determined to be 4.12 eV, and that of ITO layer is 5.11 eV, by extrapolating the linear section of (*αhν*)^2^ to the *hv* axis. The large band gap means that the absorption of ITO/Ga_2_O_3_/Ag/Ga_2_O_3_ multilayer in UV range is smaller than that of 47 nm ITO layer. Table 4 shows the transmittance at 365 nm and sheet resistance of sample 4 and 47 nm ITO. The sample 4 has a reduction in sheet resistance compared to the 47 nm ITO sample. The transmittance of sample 4 is higher than that of 47 nm ITO and other reported metal-doped ITO [12,13,14,15]. These results reveal that the ITO/Ga_2_O_3_/Ag/Ga_2_O_3_ multilayer exhibits an advantage of transmittance at UV range and conductivity.

**Figure 6:**
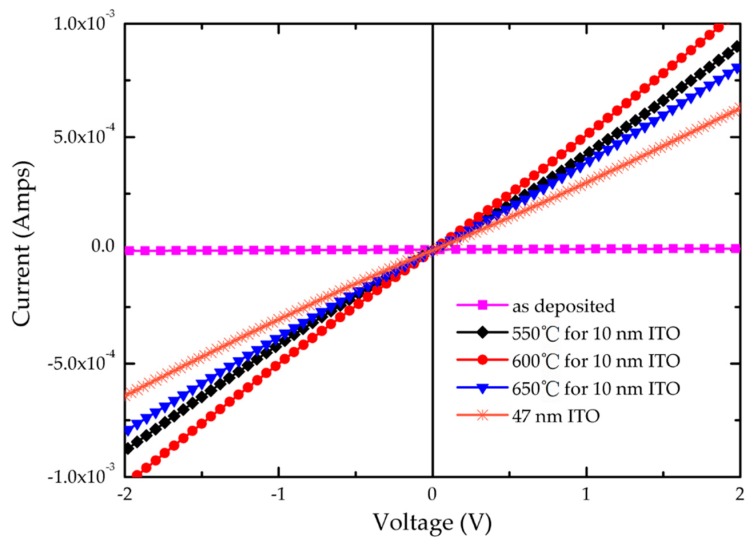
Ohmic contact characteristics of ITO/Ga_2_O_3_/Ag/Ga_2_O_3_ multilayer with different annealing temperature for ITO contact layer.

Finally, we prepared a series of ITO/Ga_2_O_3_/Ag/Ga_2_O_3_ multilayers on AlGaN-based UV epitaxy to study the specific contact resistance through the CTLM model. These samples were fabricated in the same process as sample 1 and sample 3, 4, 5. The 10 nm ITO contact layer was annealed at 550/600/650 °C respectively, and then the whole ITO/Ga_2_O_3_/Ag/Ga_2_O_3_ multilayer was annealed at 600 °C. The annealing process of ITO and the ITO/Ga_2_O_3_/Ag/Ga_2_O_3_ multilayer both maintained in N_2_/O_2_ (200 sccm:35 sccm) mixture ambient for 1 min. As reference, a 47 nm ITO was also deposited on epitaxy and annealed at 600 °C in N_2_/O_2_ (200 sccm:35 sccm) mixture ambient for 1 min. Figure 6 shows the Ohmic contact characteristics of annealed ITO/Ga_2_O_3_/Ag/Ga_2_O_3_ multilayer with p-GaN measured by Electroluminescence system and CTLM mode. The I–V characteristics of the as-deposited ITO/Ga_2_O_3_/Ag/Ga_2_O_3_ multilayer are insulated, because the Ga_2_O_3_ films have the properties of non-diffusion of metals, poor conductivity and insulation on the p-GaN surface. However, the multilayer whose ITO was annealed in nitrogen-oxygen atmosphere at 550 °C /600 °C /650 °C shows linear I–V characteristics on the surface of p-GaN. Also, all the annealed ITO/Ga_2_O_3_/Ag/Ga_2_O_3_ multilayers exhibit higher current compared to the 47 nm ITO on p-GaN. The slope of 600 °C annealed I-V curve is highest. The specific contact resistance of 600 °C annealed sample could reach 2.36 × 10^−3^ Ω·cm^2^. In contrast, the specific contact resistance of 47 nm ITO on AlGaN-based UV epitaxy is 5.68 × 10^−3^ Ω·cm^2^.

**Figure 7:**
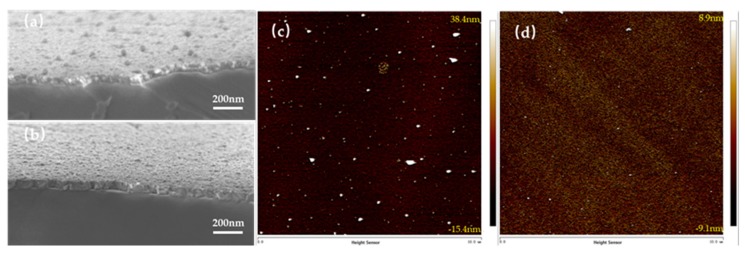
The surface morphology measured by SEM and AFM. (**a**,**c**) are for ITO/Ga_2_O_3_/Ag/Ga_2_O_3_ multilayer on AlGaN-based UV epitaxy after annealing at 600 °C. (**b**,**d**) are for 47 nm ITO on AlGaN-based UV epitaxy after annealing at 600 °C.

To further compare the differences between ITO/Ga_2_O_3_/Ag/Ga_2_O_3_ multilayer and the 47 nm ITO, we measured the surface morphology using scanning electron microscope (SEM) and AFM. The Figure 7a,b show SEM micrographs of the ITO/Ga_2_O_3_/Ag/Ga_2_O_3_ multilayer and 47 nm ITO on the AlGaN-based UV epitaxy. The surface of 47 nm ITO is smoother than that of the ITO/Ga_2_O_3_/Ag/Ga_2_O_3_ multilayer. Besides, the thickness of the multilayer is about 48 nm measured by SEM cross-section micrograph. The root-mean-square (RMS) surface roughness of the ITO/Ga_2_O_3_/Ag/Ga_2_O_3_ multilayer and 47 nm ITO on a 10 × 10 μm^2^ area are 6.92 nm and 2.36 nm respectively measured by AFM. A rough surface is beneficial for light emitting form chips to external. The rougher surface of the ITO/Ga_2_O_3_/Ag/Ga_2_O_3_ multilayer may be another reason for its higher transmittance.

## 4. Conclusions

In this paper, a complex transparent conductive electrode based on Ga_2_O_3_ for AlGaN-based UV LEDs is proposed. The complex transparent conductive electrode consists of a 10 nm ITO, a 15 nm Ga_2_O_3_, a 7 nm Ag, and a 15 nm Ga_2_O_3_, forming a ITO/Ga_2_O_3_/Ag/Ga_2_O_3_ multilayer. The ITO/Ga_2_O_3_/Ag/Ga_2_O_3_ multilayer was grown by magnetron sputtering. The resistance and transmittance ITO/Ga_2_O_3_/Ag/Ga_2_O_3_ multilayer with a different annealing temperature was studied and analyzed systematically. With suitable annealing conditions, the ITO/Ga_2_O_3_/Ag/Ga_2_O_3_ multilayer reaches a 92.8% transmittance at 365 nm and a specific contact resistance of 2.36 × 10^−3^ Ω·cm^2^. The XPS and AES results show that the diffusion of Ag in the multilayer leads to a low sheet resistance of the ITO/Ga_2_O_3_/Ag/Ga_2_O_3_ multilayer. The reason for the high transmittance of the ITO/Ga_2_O_3_/Ag/Ga_2_O_3_ multilayer in the UV range is the 5.11 eV band gap. These situations provide the improvement in optical characteristics of 365 nm UV LEDs. These results indicate that the proposed ITO/Ga_2_O_3_/Ag/Ga_2_O_3_ multilayer is a promising alternative for TCE to further improve the optical and electrical performances of AlGaN-based UV LED.

## Figures and Tables

**Table 1:** Sheet resistance of a Ga_2_O_3_/Ag/Ga_2_O_3_ multilayer on quartz substrates at different annealing conditions.

No.	Annealing Temperature	Annealing Ambient	Annealing Time	Sheet Resistance (Ω/sq)
1	As deposited	As deposited	As deposited	23.86
2	400 °C	N_2_ 200 sccm:O_2_ 35 sccm	1 min	32.1
3	500 °C	N_2_ 200 sccm:O_2_ 35 sccm	1 min	27.74
4	600 °C	N_2_ 200 sccm:O_2_ 35 sccm	1 min	16.45
5	600 °C	O_2_ 35 sccm	3 min	30.6
6	600 °C	O_2_ 100 sccm	1 min	40.93

**Table 2:** XPS data of Ga_2_O_3_/Ag/Ga_2_O_3_ multilayer on quartz substrates.

Name	As Deposited	Annealing	As Deposited	Annealing	As Deposited	Annealing	As Deposited	Annealing
	Peak BE		Height CPS		FWHM eV		Atomic %	
Ag3d	367.24	367.06	13,581.03	6651.81	0.95	0.95	0.47	0.29
O1s	530.89	530.7	224,669.16	265,423	2.04	1.7	50.67	50.97
Ga2p_3_	1118.21	1117.93	705,296.57	772,390.77	1.66	1.62	34.31	35.62

**Table 3:** The transmittance and sheet resistance of ITO/Ga_2_O_3_/Ag/Ga_2_O_3_ multilayer on quartz substrates.

Sample	Annealing Temperature		Sheet Resistance	Transmittance at 365 nm
	10 nm ITO	ITO/Ga_2_O_3_/Ag/Ga_2_O_3_		
1	No annealing	No annealing	386.7 Ω/sq	48.04%
2	No annealing	600 °C	164.0 Ω/sq	69.35%
3	550 °C		36.9 Ω/sq	80.31%
4	600 °C		20.1 Ω/sq	92.68%
5	650 °C		48.1 Ω/sq	72.09%

**Table 4:** Transmittance at 365 nm and sheet resistance of sample 4 and 47 nm ITO on quartz substrates.

Sample	47 nm ITO	Sample 4
Transmittance at 365 nm	79.15%	92.68%
Sheet resistance	57.63 Ω/sq	20.1 Ω/sq
